# W. H Atkinson, M.D., D.D.S.

**Published:** 1891-06

**Authors:** 


### W. H ATKINSON, M.D., D.D.S.

Space did not permit an extended notice of Dr. Atkinson's life-work in the May issue of this journal, nor had we the full data necessary. This has since been received through the kindly efforts of several friends, to whom our thanks are due.

Dr. Atkinson was born January 23, 1815. His father was a Methodist minister and his mother was of Quaker origin. She was the doctress of the neighborhood in Mercer County, then very sparsely settled. This gave the subject of this sketch a natural inclination towards medicine, although at an early age he was apprenticed to a tailor, filling up spare time with farm-work.

He early exhibited a desire to investigate scientific problems, and at a later period he went to Meadville, Pa., to study medicine with Dr. William Woodruff, whose daughter, Martha C., became his wife May 17, 1840. He graduated as an M.D. at Willoughby University, Willoughby, Ohio, in 1847, his thesis being upon "Sleep." This college was subsequently burned with its records, so that this early effort cannot be compared with his later work.

He began the practice of medicine and especially surgery at Meadville, in partnership with his preceptor, his father-in-law. He removed from here to Norwalk, Ohio, where he practised as a surgeon and physician. While very successful in these related branches he saw a wide field opening in dentistry, then in its infancy, and again changed his residence to Cleveland, Ohio, in 1853, where he entered into partnership with Dr. Slawson. At this time he took Dr. Charles R. Butler as student, and subsequently formed a partnership with him.

He received the dental degree at the Ohio College of Dental Surgery at Cincinnati, 1859, and removed to New York in 1861, where for a year he managed the S. S. White Dental Depot.

Dr. C. R. Butler writes of him in connection with his Cleveland experience: "Dr. Atkinson conceived that dentistry must be far more than a trade, and from that time he strove to make it a great and ennobling profession. He came to Cleveland full of vigor and enthusiasm, and soon became known as one of the most aggressive and progressive members of the dental profession. He was not one to be led, nor was he satisfied with an inferior rank. He was an earnest student in microscopy, in fact all natural sciences claimed his attention. His professional life here was a gratifying success. He was one of the very first to advocate a higher fee for dental operations. When he came to Cleveland professional remuneration was at a low standard. This he soon changed for his own good and that of his brother dentists."

He opened an office in New York in 1861, in the house of Dr. William H. Allan, 18 West Eleventh Street. The following year he moved to 41 East Ninth Street, where he remained.

He founded the First District Dental Society, and devoted a large portion of his time to advance the status of dentistry.

He was the first president of the American Dental Association, and was instrumental in organizing the New York College of Dentistry.

His wife nobly co-operated with him not only in entertaining his many professional visitors, but attended the conventions with him and shared his interest and ambition to place the profession upon a higher level.

He was a member of many societies. Honorary member of the

Odontological Society of New York, Central Dental Society of Northern New Jersey, California State Dental Association. He was an active member of the First District Dental Society of New York, having been at one time its president. He was also a member of the American Microscopical Society, Odontographic Society of Pennsylvania, and other organizations.

About a year ago Dr. Atkinson had a severe attack of *la grippe*, and never fully recovered his vigor. Three weeks before his death a persistent bronchitis set in, which was followed by pneumonia, terminating fatally at 9.15 P.M., Thursday, April 2, 1891. His constant thought for professional advancement was indicated in the last intelligible conversation had with him, when he mentioned the fact that he had engaged to write certain papers, but had not been able to commence them on account of continued indisposition.

His family consisted of three sons and five daughters, of whom are living one son, Dr. Charles B. Atkinson, who has been his father's partner in practice.

Of the five daughters, three married dentists,—Dr. George Viall, Pasadena, Cal.; Dr. Thomas Rowe, Cobourg, Canada, and Dr. John M. Crowell, deceased.

Dr. Atkinson was buried in the family plot at Woodlawn Cemetery.

